# Apolipoprotein E Mimetic Peptide-Mediated Modulation of Lipid Homeostasis

**DOI:** 10.3390/biom16091352

**Published:** 2026-09-17

**Authors:** Dilan R. Patel, Cole L. Martin, G. M. Anantharamaiah, Stephen G. Aller

**Affiliations:** 1 Department of Biochemistry and Molecular Genetics, University of Alabama at Birmingham, Birmingham, AL 35294, USA; 2 Department of Clinical and Diagnostic Science, University of Alabama at Birmingham, Birmingham, AL 35294, USA; 3 Department of Medicine, University of Alabama at Birmingham, Birmingham, AL 35294, USA; ganantha@uabmc.edu

**Keywords:** peptide, cholesterol, hypercholesterolemia, atherosclerosis, apo E, LDL

## Abstract

Lipid homeostasis is essential for maintaining cellular function and metabolic balance in all organisms, and its dysregulation can contribute to hypercholesterolemia, atherosclerosis, and cardiovascular disease. Adverse health outcomes are associated with elevated levels of low-density lipoprotein cholesterol (LDL-C), retention of which causes macrophages to transition into foam cells, leading to widespread inflammation, cardiovascular disease, and atherosclerosis. Statins (HMG-CoA reductase inhibitors) are considered the gold standard for LDL and very-low-density lipoprotein (VLDL) reduction, yet they exhibit side effects like myalgia and insulin resistance. Apolipoprotein E (apo E)-based lipid-binding synthetic mimetic peptides have been shown to clear cholesterol from the blood through canonical hepatic pathways such as HSPG receptors and are currently being studied to reduce plasma cholesterol. This review examines the development, biological properties, and therapeutic potential of the apo E mimetic peptide AEM-28. AEM-28 reduces atherogenic lipoproteins from plasma efficiently in multiple preclinical models as well as Phase I clinical trials in humans, where general tolerance of the treatment was observed. AEM-28 utilizes a unique mechanism of action that involves binding to atherogenic lipoproteins with multiple receptor-binding domains, facilitating enhanced clearance via the highly abundant heparan sulfate proteoglycans on hepatocytes. These attributes support the potential of AEM-28 as a complementary therapeutic or even an alternative for patients requiring LDL apheresis and those with hypercholesterolemia, atherosclerosis, or cardiovascular disease.

## 1. Introduction

Dyslipidemia is often characterized by a change in plasma lipid levels that poses an increased risk for cardiovascular disease [1]. Hypercholesterolemia, a form of dyslipidemia characterized by elevated LDL levels, affects approximately one-third of individuals worldwide [1]. Diagnostically, severe hypercholesterolemia is presented as LDL levels of 190 mg/dL or higher [2].

Cholesterol biosynthesis occurs within the Endoplasmic Reticulum (ER) of hepatic cells through a series of enzyme-dependent reactions [3]. Once synthesized, it is transported to the bloodstream or made into bile [3]. One important function of cholesterol is to maintain membrane fluidity within all cells in an organism [3]. Additionally, cholesterol is the starting material for many biomolecules, such as Vitamin D, bile acids, and hormones such as testosterone [4].

Cholesterol is primarily circulating as part of three lipoproteins: very-low-density lipoprotein (VLDL), low-density lipoprotein (LDL), and high-density lipoprotein (HDL). LDL is derived from VLDL catabolism in the liver to export cholesterol to other cells via secretion into the plasma [5]. Once in circulation, LDL binds to an LDL receptor and gets internalized within the cells, where it gets broken down by lysosomal enzymes [5]. LDL has a variety of lipids, with its primary structural protein comprising apo B-100 [5]. Apo B-100 is also a constituent of VLDL [6].

VLDL is both synthesized and secreted from the liver, carrying phospholipids, triglycerides, and cholesterol [7]. The lipoprotein is composed of multiple apolipoproteins such as apo B-100, apo C-I/II, and apo E [7]. Apo E is a component of VLDL. Apo E mediates binding to hepatic cells via the abundantly available Heparin Sulfate Proteoglycan Receptors (HSPG), LDL receptor (LDLR), and LDL-R related protein (LRP) [8]. Isoforms of apo E have been associated with various disease states. Particularly, apo E-2 has been associated with an increased risk of hyperlipidemia and hypercholesterolemia [9,10]. Other isoforms such as apo E-4 have been associated with Alzheimer’s disease [9,10].

While high levels of LDL and VLDL are involved in increasing lipid-mediated inflammatory processes, high levels of HDL are considered anti-inflammatory and anti-atherogenic. HDL is involved in transporting cholesterol back from peripheral tissues to the liver via the reverse cholesterol transport pathway (RCT) [11]. This mechanism of cholesterol transport is crucial since the excess cholesterol that is transported back to the liver is either metabolized into bile acids/salts or directly excreted as bile [11]. Apolipoprotein (apo) A-I is the major protein component of HDL [12]. Apo A-I is not only responsible for the structural integrity of the HDL particle, but also interacts with downstream enzymes, such as the plasma enzyme Lecithin–Cholesterol Acyltransferase or LCAT, which converts free cholesterol into cholesteryl ester [12,13], a step in RCT.

The balance between LDL, VLDL, and HDL in the blood is critical because of the downstream consequences of elevated LDL and VLDL. Abnormally high amounts of LDL within the blood can be induced by diet, a lack of exercise, genetic conditions, and lifestyle choices [14]. In the presence of excess oxidized LDL, which causes cellular stress, various immune cells are recruited to the area, such as macrophages [15]. These macrophages are induced to a pro-inflammatory M1 phenotype via interferon-γ produced by either mast cells or T-Helper 1/17 cells [16]. The M1 macrophages start to ingest the oxidized LDL and degrade the lipoprotein by lysosomal digestion [16]. Over time, macrophage-mediated cholesterol efflux decreases, increasing cellular oxidized lipid concentration, promoting foam cell development [16]. Furthermore, new research has shown that the heterogeneity of both innate and adaptive immune cells are participants within the progression of atherosclerosis [17,18,19].

Additionally, the secretion of inflammasomes may have some mechanism or interaction that starts foam cell development [20]. Removal of oxidized lipids from LDL is important for limiting monocyte recruitment and foam-cell formation [15]. Foam cells exit the area slowly compared to normal macrophages free of lipid load, and undergo apoptosis, accumulating cholesterol, which eventually results in plaque formation [15]. The process of reducing cellular macrophages is a key step in inhibiting atherosclerotic plaque formation [15,21].

HDL function exhibits both antioxidant and anti-inflammatory properties that aid in preventing cardiovascular and inflammatory diseases [22]. HDL can remove cholesterol from macrophages via the RCT, thus inhibiting their transition to foam cells [23]. This reduces inflammation and decreases the progression of atherosclerosis. HDL can inhibit LDL oxidation, which prevents oxidative stress experienced by cells locally [14,16].

While HDL exhibits protective properties, it alone does not decrease the risk of mortality from inflammatory diseases. Diet and exercise are the first modes of therapy employed to manage blood cholesterol concentration. Current therapeutics for high cholesterol are statins, ezetimibe (NPC1L1 inhibitor), and Proprotein Convertase Subtilisin/Kexin type 9 (PCSK9) inhibitors. For inflammatory diseases, therapeutics are being studied that target cytokines CRP, IL-1β, IL-6, IFN-γ, and TNF-α produced from foam cells during atherosclerosis [24]. While these therapeutics have been the standard of care, they do carry drawbacks, such as muscle fatigue and others discussed further in Section 1, allowing for the development of the next generation of therapeutics [24,25].

A potential addition to the new generation of therapeutics is Apolipoprotein E Mimetic-28 (AEM-28), a 28-residue peptide-based approach to control hypercholesterolemia and dyslipidemia [26]. AEM-28 has been shown in vitro, in vivo, and in human trials to lower plasma cholesterol. The purpose of the review is to compile the research that supports the ability of AEM-28 to lower plasma cholesterol via a unique mechanism of action on atherogenic lipoproteins and exhibit protective effects on cells such as macrophages. This research has gone from the fundamentals of peptide design to mimic the properties of apo E, using cell systems to dyslipidemic mouse, rabbit, and monkey models and to the completion of Phase I trials (NCT02100839). A review of this category is not currently available in the literature that highlights the protective effects of apo E mimetics via its unique mechanism of action on decreasing atherogenic lipoproteins.

## 2. Current Therapeutic Strategies for Hypercholesterolemia and Their Limitations

The current therapeutic options that are available vary in their mechanism of action, LDL reduction, anti-inflammatory effects, and their dose limits. Table 1 shows these categories in each of the therapies mentioned in Section 1.

Statins are currently considered the gold standard option for reducing cholesterol or LDL levels in individuals suffering from hypercholesterolemia. Statins work by inhibiting the enzyme HMG-CoA reductase, which is involved in cholesterol biosynthesis [31]. HMG-CoA is a metabolite that is a part of the multistep biosynthesis of endogenous cholesterol [31]. First published by Akira Endo in 1976, Compactin came to be the first statin identified [31]. While the initial clinical trials to investigate its safety and efficacy were promising, research was halted after discovering that high doses of Compactin were associated with lymphomas in dogs [31]. This led to investigators researching structurally similar compounds, which led to the discovery of Lovastatin, which became the first statin on the market in 1987 after FDA approval [31].

**Figure 1:**
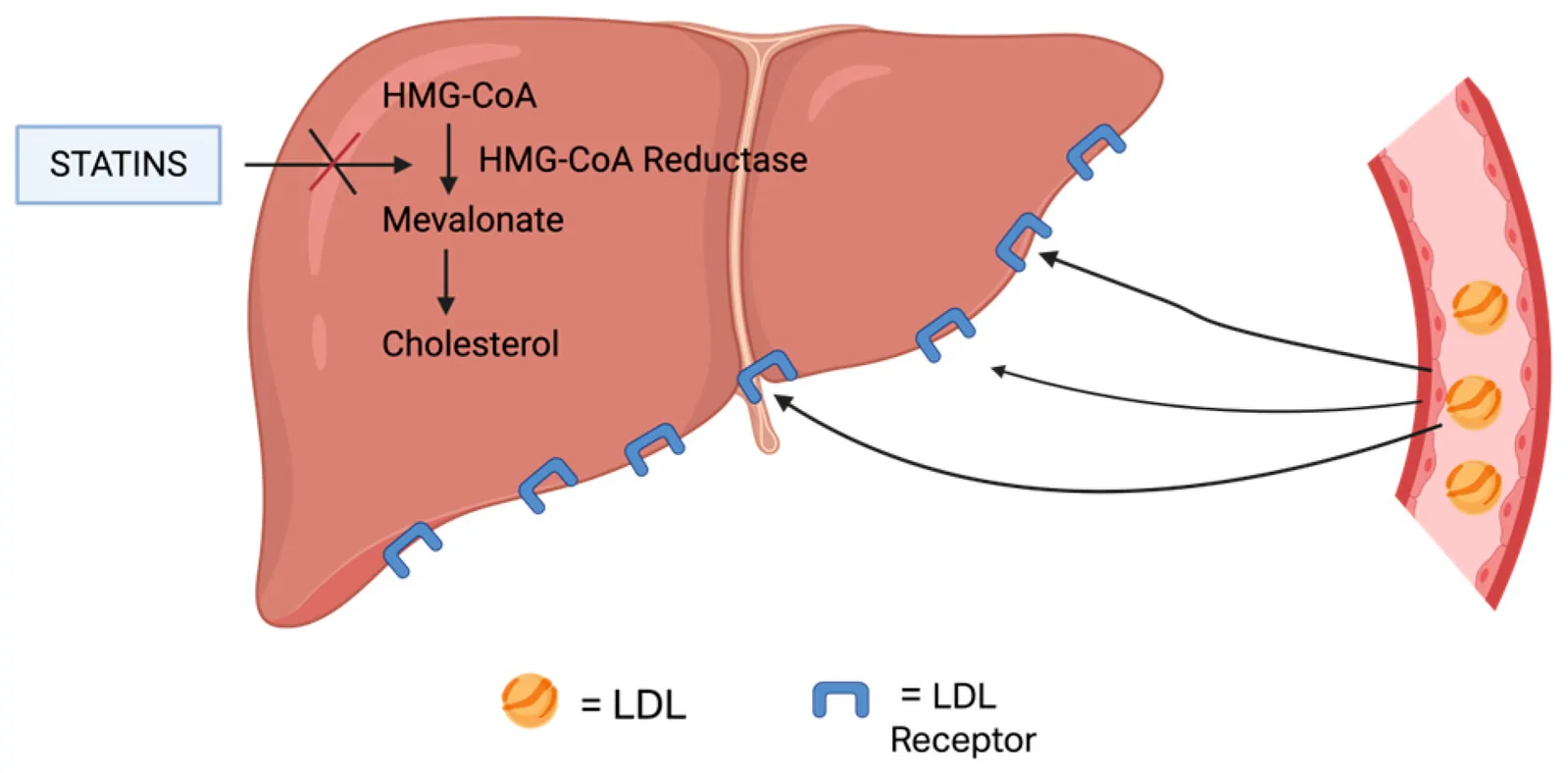
General mechanism of statins. By inhibiting HMG-CoA reductase (arrow), cholesterol biosynthesis is stopped, allowing LDL receptors to stay active longer and internalize plasma cholesterol via LDL. Created in BioRender. Patel. (2026). https://BioRender.com/.

Statins inhibit cholesterol biosynthesis early within the reaction mechanism of endogenous cholesterol biosynthesis, reducing the levels of LDL cholesterol in the bloodstream (Figure 1) [32]. When cholesterol levels are high, the levels of HMG-CoA reductase and LDL receptors are decreased, leading to higher levels of LDL in the blood, increasing the chances of cardiovascular disease [33]. Meanwhile, the inverse of this is observed, where low levels of LDL cholesterol increase the levels of both HMG-CoA reductase and LDL receptors [33]. Thus, when statins inhibit cholesterol biosynthesis, low levels of cholesterol are present in hepatic cells [34]. This results in increased amounts of hepatic LDL receptors, thus clearing large amounts of LDL from the plasma [34].

Statins not only reduce plasma cholesterol but also carry other effects such as being able to reduce the progression of coronary lesions [34]. In the ASTEROID trial, rosuvastatin led to plaque regression in 64% of participants following statin treatment [34]. However, some statins, such as Pravastatin dosed at 80 mg for 18 months, showed progression of coronary atherosclerosis [34]. Another mechanism in which statins mitigate atherosclerotic progression is by stabilizing the plaque [34]. This is accomplished by an increased amount of calcified plaque while the amount of non-calcified plaque is reduced [34,35]. How this may help with plaque stabilization is still unknown. However, Almeida et al. suggest that statins mediate the M2 macrophage phenotype, which is associated with macrocalcification, helping with plaque stabilization [34]. Additionally, atherosclerosis promotes an inflammatory environment near the plaque site. The chemokines and cytokines that are produced by foam cells are associated with an increase in inflammation [34,36]. Statins have been shown to reduce these signals and reactive oxygen species [34,36].

While statins have been the number one treatment option for lowering cholesterol in those who suffer from dyslipidemia, there are drawbacks associated with the use of statins. Potential mechanisms for how Statin-Associated Muscle Symptoms (SAMSs) occur are still being identified [37]. Ruscica et al. propose that statins are major antagonists of chloride channels located on muscle cell membranes, which may lead to contraction [37]. SAMSs also occur through the impairment of membrane potentials of mitochondria [37]. These impairments lead to a decrease in maximum oxygen intake and ATP production within mitochondria [37]. This was further compounded by muscle biopsies showing mitochondria with increased stores of lipids in patients treated with statins [37]. Drug–drug interactions may also play a role in the causation of SAMSs [37]. Patients carry a variation of cytochrome P450 (CYP) 3A4, which is responsible for drug metabolism within the body, and when patients take multiple medications, there is a chance that there may be interactions with the CYP pathway, increasing statin exposure [37]. Additionally, response may be suboptimal in individuals in terms of utilizing a statin endogenously. This is characterized as having less than 40% reduction in baseline LDL levels while on statins within a 2-year timeline [38]. In a prospective cohort study in Nottingham, UK, they observed that out of 165,411 patients, over 51% were considered suboptimal [38]. These suboptimal responders were significantly more likely to have an incident cardiovascular disease event [38]. For these suboptimal responders, a mechanism that bypasses the LDL-R bottleneck may be clinically necessary. Statin efficacy has been recorded in the literature; however, statins carry inherent disadvantages that may deter patients from their use.

### 2.1. PCSK9 Inhibitors

PCSK9 was first discovered by Seidah and colleagues in Montreal [39]. PCSK9 is a proteolytic enzyme that regulates the amount of LDL receptors found on the surface of hepatic membranes [40]. Regulation is mediated by PCSK9 binding to LDL receptors, allowing for the complex to be endocytosed and ultimately degraded by lysosomes [40]. This regulates both intracellular cholesterol levels in hepatocytes and blood LDL levels [40]. Those who possess mutations that lead to gain-of-function in PCSK9 have elevated LDL levels similar to those with familial hypercholesterolemia (FH) [40]. For those who suffer from FH, high intracellular cholesterol causes upregulation of PCSK9, which decreases the amount of LDL receptors and thus increases circulating LDL levels [39,40].

**Figure 2:**
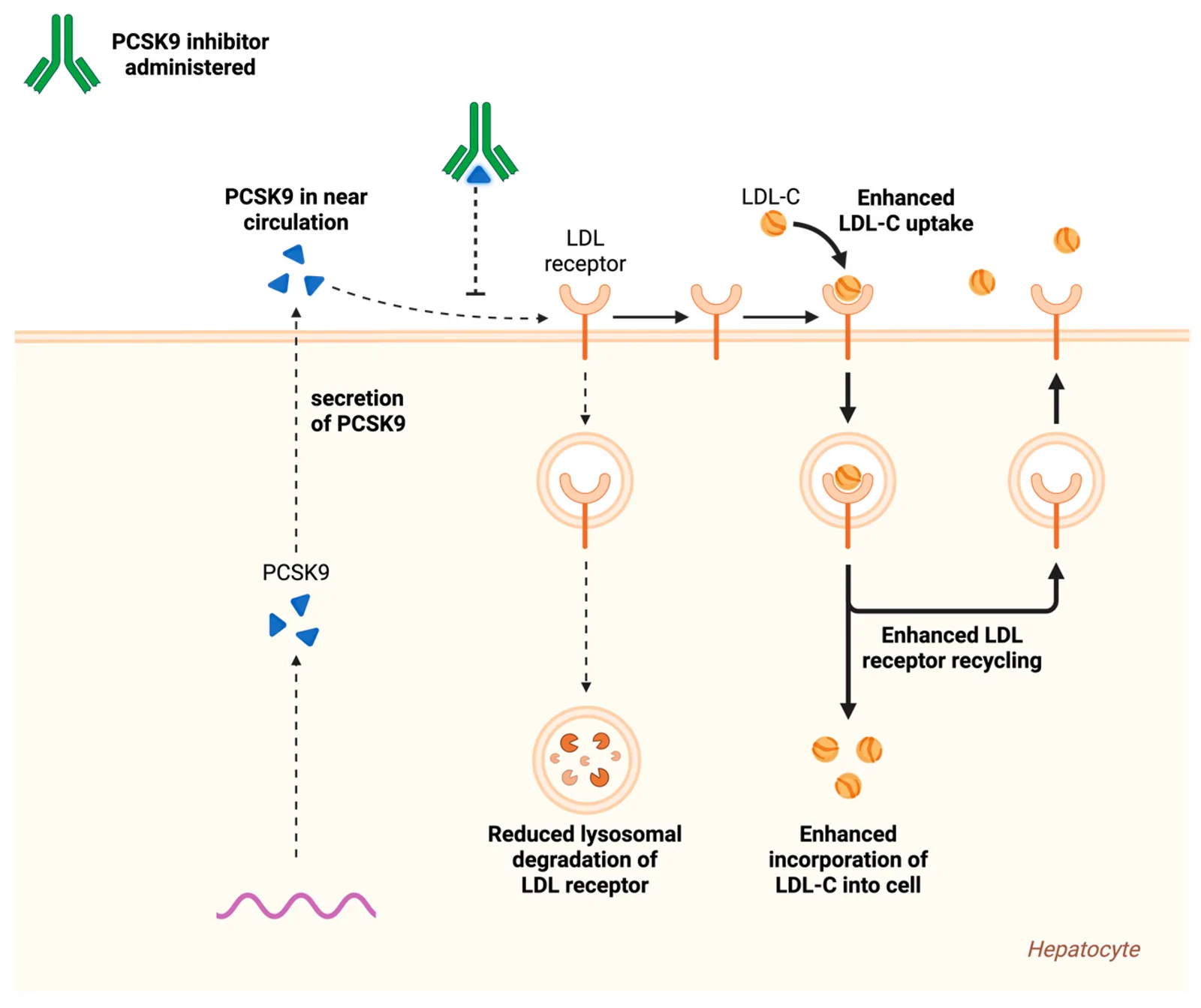
General mechanism of action for PCSK9 inhibitors. PCSK9 inhibitors inhibit binding of PCSK9 to the LDL receptor, thus decreasing the degradation of the LDL receptor. This allows for the LDL receptor to stay active for longer, clearing atherogenic lipoproteins from the plasma. Created in BioRender. Patel (2026). https://BioRender.com/.

These observations led to the development of PCSK9 inhibitors such as alirocumab and evolocumab [40]. These two inhibitors are human monoclonal antibodies that bind free PCSK9, preventing premature internalization and degradation of LDL receptors, allowing for more LDL receptors and thus increased LDL clearance (Figure 2) [40]. These antibodies are highly specific for PCSK9 and decrease the chances of drug–drug interactions [40]. These inhibitors are given through subcutaneous injection and reduce PCSK9 levels 4–6 h after the injection period [40]. Both antibodies have been shown to decrease LDL levels, as seen in Table 1.

PCSK9 can enhance atherosclerosis by decreasing the amount of LDL receptors, which increases the amount of LDL in circulation [41]. Research has also shown that PCSK9 is involved in pro-inflammatory pathways due to its unique cysteine-rich domain found on its C-terminus, which is structurally similar to resistin [41]. Resistin is known to bind and activate toll-like receptor 4 (TLR4), activating numerous cytokine cascades [41]. PCSK9 inhibitors have been shown to stabilize atherosclerotic plaque and reduce inflammation [42]. Several biomarkers associated with atherosclerosis decrease after the start of PCSK9 inhibition, such as Osteopontin, Osteoprotegerin, and Metalloprotease-9 [42].

Drawbacks and adverse events associated with PCSK9 inhibitors include injection site reactions, upper respiratory tract infections, and adherence [43]. In the ODYSSEY LONG TERM trial, patients received a combination therapy of statins and PCSK9 inhibitors like alirocumab [43]. Patients reported events such as injection site reactions, myalgia, and neurocognitive events [43]. Another drawback of PCSK9 inhibitors is their cost, which is around $14,100 per year, higher than generic statins [43,44]. This proves difficult for individuals seeking non-statin-based therapies, as these therapies do not meet incremental cost-effectiveness thresholds and increase health care costs [43]. While PCSK9 inhibitors demonstrate efficacy in reducing plasma cholesterol, drawbacks, such as injection site reactions and a lack of adherence due to high treatment cost, could contribute to the lack of usage of the therapeutic.

Other PCSK9 inhibitors have recently come to market, such as Inclisiran. While it targets the PCSK9 protein, its mechanism of action is quite different from antibody-derived therapeutics. Inclisiran is a siRNA-based therapeutic, which targets the mRNA of PCSK9 and degrades it via the RISC complex [45]. This allows LDL-R to remain in the cellular membrane much longer and clear LDL from the plasma [45]. The formulation of Inclisiran has allowed it to specifically target the liver by the addition of synthetic N-acetylgalactosamine [45]. This is due to the sugar’s nature, which is complimentary to asialoglycoprotein receptors found on hepatocytes [45]. The dosing of Inclisiran is an injection on day one, repeated on day 90, then followed every 6 months [46]. This is one of the hallmark differences between Inclisiran and antibody-based therapeutics targeting PCSK9. Inclisiran is only injected every 6 months after months 1 and 3, whereas antibody-based therapeutics are injected every 2–4 weeks [40,46]. Multiple Phase III trials have demonstrated Inclisiran’s efficacy in lowering LDL levels [46]. Adverse effects were observed during Inclisiran’s Phase I trials, such as mild self-limiting rash at the injection site, musculoskeletal pain, cough, headache, hyperpigmentation, and back pain [45]. Inclisiran inhibits PCSK9 by silencing its mRNA, reducing LDL levels to a similar capacity to antibody-based PCSK9 inhibitors, and decreasing drug administration. The exhibited therapeutic side effects may contribute to its lack of use in patients.

### 2.2. Other Therapies

While statins and PCSK9 inhibitors are the main options for cholesterol reduction, alternatives are available. One such alternative is ezetimibe, which works by reducing the delivery of chylomicrons and cholesterol to the liver via the small intestines [47]. Common side effects include upper respiratory tract infection, sinusitis, and arthralgia [47]. Bile acid sequestrants are another therapy which base their mechanism of action on binding bile acids, which inhibits absorption into the body [47]. This reduces the amount of bile acid, which promotes the conversion of cholesterol to bile acids, decreasing the amount of cholesterol [47]. The adverse effects of these sequestrants include constipation [47]. The most rigorous of alternative therapies is lipoprotein apheresis. Apheresis is usually done biweekly and takes around 2 h over a prolonged time frame [47,48]. While effective in removing harmful lipoproteins and associated molecules, the necessity of its constant usage is quite detrimental to quality of life.

## 3. Mechanism of Cholesterol Clearance by Apo E Mimetic Peptides

AEM-28 is a 28-amino-acid-residue peptide designed to mimic apo E in its ability to clear plasma cholesterol. The peptide comprises an apo A-I mimetic platform called 18A (DWLKAFYDKVAEKLKEAF) covalently linked to the receptor-binding domain of apo E (residues 141–150, LRKLRKRLLR) [49]. To fully understand the ability of AEM-28 in reducing plasma cholesterol and acting as an anti-inflammatory agent, it is best to discuss its primary components and their role in cholesterol clearance.

**Figure 3:**
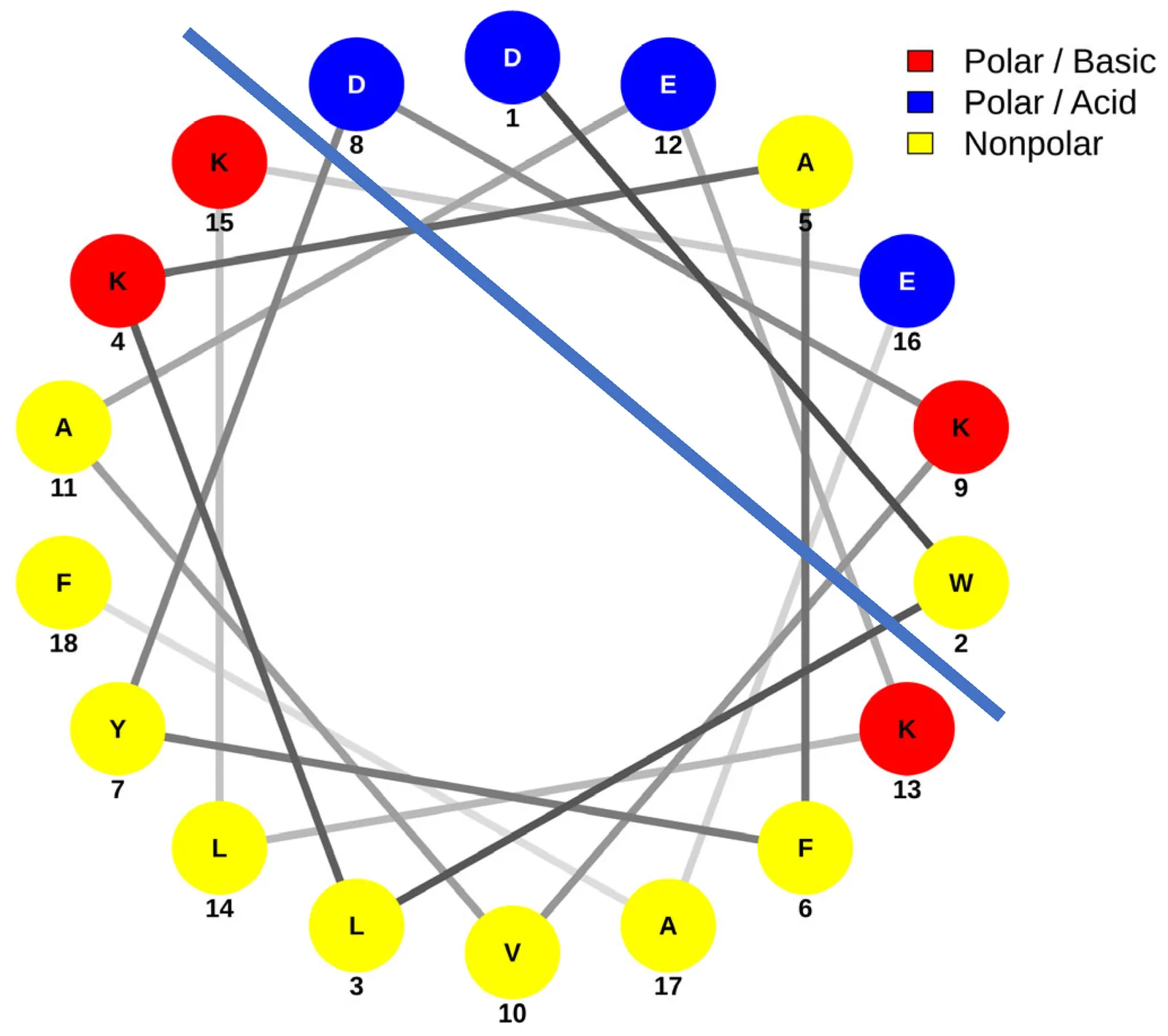
Helical wheel representation of 18A. The lines connecting each residue represent the peptide chain starting from residue 1 to residue 18. Additionally, the blue line intersecting the wheel demonstrates the polar/nonpolar interface of 18A. The helical model of 18A illustrates key characteristics of a class A helix, such as its amphipathic nature with polar and nonpolar sides and the polar/nonpolar interface that allows for lipid interaction. The figure was generated using the web application NetWheels.

The first component of AEM-28 is 18A (Ac-18A-NH_2_), an apo A-I mimetic peptide that comprises 18 amino acids sharing no sequence homology with apo A-I or any other apolipoproteins [50,51]. However, it does share the ability to bind lipids and form peptide–lipid complexes that are similar in shape and size to those formed by human apo A-I [50]. The class A amphipathic helix is a structural motif found in apo A-I and other apolipoproteins involved in lipid interactions/binding [51,52]. It is characterized by basic amino acids near polar–nonpolar interfaces with acidic amino acids at the center of the polar face [51,52]. The presence of positively charged residues with long acyl chains at the polar–nonpolar interface enables the peptide backbone to bury deeply into the lipid membrane, enhancing lipid-binding efficacy (Figure 3) [52]. Secondary structure formation and lipid-associating effects are enhanced by the amidated C-terminus and acetylated N-terminus [53]. This modification alone enhances the lipid-associating properties in that, while 18A cannot clear turbidity due to multilamellar vesicles (MLVs) of POPC, Ac-18A-NH_2_ spontaneously clears the turbidity of POPC-MLV to form peptide–lipid complexes [54].

18A and its variants were tested to evaluate their ability to lower plasma cholesterol and their effect on atherosclerotic plaque. The variants included 18A with differing amounts of Phenylalanine added to the hydrophobic face of the class A amphipathic α-helix [50]. The 18a variant, 4F, was designed by replacing the existing two Leu residues from 18A with Phe residues [50]. 4F was able to solubilize MLVs of egg phosphatidylcholine (PC), although apo A-I does not solubilize this lipid [50]. 4F demonstrated that a synthetic class A helical peptide could have an enhanced lipid binding affinity compared to apo A-I [50]. Also, when 4F was administered, it showed no change in total plasma cholesterol levels [50]. However, it increased Pre-β-HDL and antioxidant enzyme activity, such as paraoxonase-1 (PON-1), which are key components for the enhancement of RCT [50,55,56]. The peptide also showed a reduction in nascent atherosclerotic lesions but not in mature lesions [57]. This suggests that 4F is better suited as a preventative measure for reducing nascent lesion development; however, more research is needed to elucidate the lack of interaction of 4F with mature lesions [57]. While 4F does carry certain biochemical properties associated with a class A amphipathic helix, it does not by itself participate in the lowering of atherogenic lipoproteins when administered to dyslipidemic animal models [57]. These findings suggest that 18A and its derivatives need further refinement for clearing atherogenic lipoproteins in the plasma.

Further modification of 18A by covalently linking the receptor-binding domain of apo E, a protein component in VLDL, led to the design of apo E mimetics, which possess plasma cholesterol-reducing properties, unlike apo A-I mimetics. Apo E has been shown to have anti-atherogenic properties [58]. This was shown by administering it in mice that did not carry apo E genes, which possessed higher levels of plasma cholesterol and developed spontaneous atherosclerosis even under a normal diet [58]. The reverse was seen in mice when apo E was overexpressed and circulating cholesterol was dramatically reduced [58]. This supports that apo E, like apo A-I, carries atheroprotective properties that can be utilized to reduce LDL and VLDL [58]. Apo E is also a ligand for various receptors, such as the LDL receptor (LDLR), LDL receptor-related protein (LRP), and Heparin Sulfate Proteoglycans (HSPGs) [58]. This allows vast amounts of plasma cholesterol to be cleared from various dyslipidemic situations. The positively charged domains at the N-terminus of the apo E mimetic peptide (LRKLRKRLLR) mediate binding to the receptors mentioned earlier, which contain negatively charged domains and engage in strong ionic interactions [58,59,60]. The C-terminal domain carries the helical high-affinity lipid-binding domain [61]. Thus, it is suggested that hepatic clearance of atherogenic lipoproteins could be expedited via the binding of multiple copies of the apo E receptor domain to atherogenic lipoproteins by attaching the highly efficient lipid-associating peptide 18A, where the C-terminus is amidated [58].

Anantharamaiah et al. took residues 141–150 of apo E, LRKLRKRLLR, and covalently attached it to 18A with its N-terminus acetylated and C-terminus amidated [58]. The new peptide, AEM-28, combines the capabilities of a class A helix and the receptor-binding domain of apo E to potentially allow association of multiple copies of this peptide with LDL and VLDL to lower plasma cholesterol, bypassing the LDL-R pathway.

## 4. Additional Apo E Mimetics

While AEM-28 is the primary apo E mimetic discussed in this review, other peptide platforms have been created using Apolipoprotein E as a foundation. The first peptide is COG1410, developed by Laskowitz et al. [62]. Apo E is expressed significantly after a traumatic brain injury (TBI) due to apo E’s neuroprotective effects [62]. This is critical after a TBI since multiple inflammatory pathways are activated, releasing various molecules such as ROS, byproducts of nitric oxide, and cytokines [62]. Given the multiple protective effects of apo E during the recovery process after a TBI, a peptide harnessing apo E’s biochemical characteristics carries potential as a therapeutic. COG1410’s sequence takes residues from 138–149 (ASHLRKLRKRLL) of apo E and replaces residues at positions 140 and 145 with amino-isobutyric acid [62]. The full sequence of COG1410 is (acetyl-AS-Aib-LRKL-Aib-KRLL-amide) [62]. COG1410 has been shown to reduce inflammatory biomarkers, a similar hallmark ability of AEM-28; however, because the methodologies used to investigate this ability differed for both peptides, a valid comparison cannot be made [62,63,64]. A second apo E mimetic, ATI-5261, is based on the C-terminus of apo E and also the lipid-binding domain [65]. The peptide mimics class A amphipathic helix properties seen in apo A-I without any sequence homology, similar to 18A, and can increase nascent HDL and increase the antioxidant activity of PON-1 [50,55,56,65]. The sequence of the peptide (acetyl-EVRSKLEEWFAAFREFAEEFLARLKS-amide) allows for increased lipid efflux from ABCA1, increasing nascent HDL concentration within the plasma [65]. However, the peptide has not been shown to clear atherogenic lipoproteins like AEM-28 [65]. Similarly, AEM-28 has not been investigated to see if it increases ABCA1-mediated cholesterol efflux but has been shown to increase anti-inflammatory enzyme activity associated with HDL [63,66]. Both COG1410 and ATI-5261 vary in their application and disease model but show the vast capabilities of apo E and its foundation for potential therapeutics.

## 5. In Vitro Characterization of AEM-28

To experimentally verify this, Anantharamaiah et al. studied the changes in electrophoretic mobility of peptide-containing LDL. They first visualized the interaction of the new peptide with human LDL via agarose gel electrophoresis [67]. The lane containing LDL only was able to move towards the anode [67]. However, AEM-28-incubated LDL showed reduced mobility towards the anode [67]. This data suggested that AEM-28 was able to interact with human LDL, thus affecting LDL’s migration in the gel [67]. This degree of decreased migration was associated with the increased ratios of AEM-28 used, indicating a dose-dependent effect of AEM-28 on LDL [67]. However, just demonstrating a decrease in mobility does not fully support the hypothesis of the peptide interacting with the lipoprotein [67]. Structural analysis such as X-ray crystallography or electron microscopy could provide definitive evidence of direct AEM-28–LDL interactions, thus fully supporting the hypothesis regarding interaction [67].

Following this finding, the AEM-28-modified LDL was studied for uptake and degradation using fibroblasts [67]. At a constant LDL concentration, AEM-28 was able to mediate a 7-fold increase in LDL uptake in mouse embryonic fibroblasts [67]. Additionally, LDL uptake increased as LDL was increased with increased peptide-modified LDL, while AEM-28 concentration was constant, even above the K_d_ of ≈ 4 μg/mL [67]. This suggests that the pharmacodynamics of the peptide are optimal since a low concentration was needed to observe enhanced LDL clearance [67]. The pharmacodynamics of the peptide could be further supported by performing a true EC_50_ assay to investigate the necessary amount of AEM-28. However, the pharmacokinetics of the peptide have not been addressed in each model system investigated, particularly within lower-order mammals such as mice and transitioning to higher-order mammals such as monkeys.

This supports that AEM-28-mediated uptake of human LDL is nonsaturable and linear [67]. Additionally, these results suggest that there is an alternative to the LDL-R-mediated pathway involved in the uptake of AEM-28–LDL into the cell [67]. This characteristic of AEM-28-mediated uptake of human LDL is crucial because physiologically, the levels of LDL receptor are the bottleneck that prevents LDL from entering the liver once intracellular concentrations of cholesterol are elevated [67,68]. The uptake of LDL being nonsaturable and linear establishes a unique ability of AEM-28 to clear plasma cholesterol [67].

Residue substitutions were performed on the apo E ligand-binding domain, affecting the ability to interact with LDL and its uptake [67]. A variant of AEM-28 showed a 7-fold enhancement in comparison to LDL alone but showed similar results to AEM-28 [67]. This variant replaced the lysine within the LRKLRKRLLR sequence with arginine (AEM-28-R) [67]. Another variant replaced leucine at positions 4 and 9 with methionine to mimic the apo E receptor-binding domain of mice [67]. However, this showed 3.5-fold-higher uptake of LDL versus LDL alone, but the effect was to a lesser degree than AEM-28 [67]. The substitutions indicated that small changes to the charge or hydrophobicity of the binding domain can modulate the uptake of LDL [67]. Furthermore, a change in the hydrophobicity of the receptor-binding domain may modulate the binding of peptide to LDL, perhaps due to methionine oxidation, hence the decreased peptide activity.

As mentioned previously, the LDL uptake in the presence of AEM-28 was nonsaturable and linear, suggesting an alternative pathway for LDL uptake [67]. Anantharamaiah et al. investigated this in cell lines that were lacking in both LDL-R and LRP [67]. They noted that LDL uptake decreased by 65% in PEA13 cells and MEF4 cells, which lacked both LDLR and LRP [67]. The same decreased uptake of LDL was seen in MEF1 cells [67]. When these cells were incubated with peptide-modified LDL, AEM-28-mediated LDL uptake was 7-fold higher in MEF1 cells, 13-fold higher in PEA13 cells, and 19-fold higher in MEF4 cells [67]. This eliminated two receptors that apo E could bind to, indicating the possibility of clearance through the HSPG pathway [67]. Previously, apo E has been shown to clear atherogenic lipoproteins via the HSPG pathway [69]. Fibroblasts were incubated with both heparinase/heparitinase and were subjected to the uptake assay as described earlier [67]. LDL uptake void of the peptide was not affected [67]. However, when incubated with AEM-28 and AEM-28-R, uptake was decreased by 75% and 85%, respectively [67]. These results support the idea that AEM-28 primarily mediates enhanced LDL uptake via the HSPG pathway [67]. This characteristic of AEM-28, being able to remove LDL via HSPG, has not been seen in other therapies [67]. These findings support HSPG-mediated uptake as a potentially important mechanism contributing to enhanced atherogenic lipoprotein clearance [67]. However, some questions remain unanswered regarding HSPG-mediated clearance of LDL with AEM-28. The study demonstrated that the peptide-mediated clearance did not reach a saturable level. This study does not further investigate trying to reach saturation in any model systems used.

With AEM-28 having supporting evidence that it cleared lipoproteins via the HSPG pathway, Anantharamaiah et al. investigated the peptide in hepatocytes. Hepatocytes were chosen due to their important role in the uptake/clearance of plasma cholesterol, leading to the synthesis of bile [70]. Additionally, the uptake of VLDL, an atherogenic lipoprotein, is mediated by apo E via the HSPG pathway [70]. As shown previously, AEM-28 can interact and enhance the uptake of LDL [67,70]. However, interaction and clearance of VLDL still need to be studied [67]. LDL uptake in HepG2 cells was completed in the same manner as Datta et al. [67]. They showed that AEM-28 again enhanced the uptake of LDL 5-fold and increased degradation 2-fold in HepG2 cells [70]. Again, they showed that this effect was highly diminished with the treatment of heparinase/heparitinase [70]. This supported that AEM-28 mediates the uptake of LDL in HepG2 cells in the same manner as seen in fibroblasts [67,70]. Additionally, the peptide interaction with VLDL was analyzed [70]. Human VLDL was incubated with increasing ratios of AEM-28 from 1:0.15 to 1:0.30 [70]. Agarose gel electrophoresis was performed to visualize peptide interaction with VLDL [70]. The gel showed a decrease in intensity for bands associated with apo E, while bands associated with apo B displayed no change in intensity [70]. This suggested that apo E was being displaced by the peptide [70]. This effect was also seen when the ratio of the peptide and VLDL increased to 1:1. Again, the band corresponding to apo E had a decreased intensity compared to the VLDL control [70]. Moreover, VLDL uptake and degradation within HepG2 cells were increased via AEM-28 mediation 6-fold and 3-fold, respectively [70]. This suggested that AEM-28 displaces apo E on VLDL and adds multiple HSPG-binding domains, explaining the increased uptake of VLDL [70]. With cells treated with heparinase/heparitinase, uptake and degradation were noticeably decreased since the HSPG pathway was blocked [70]. A similar assay was performed with VLDL, which was subject to trypsin digestion to remove apo E from the lipoprotein [70]. A 15-fold increase in VLDL without apo E uptake was recorded, with the degradation noted as only a 2-fold increase [70]. AEM-28 was shown to displace apo E from VLDL but also showed more effective binding to HSPGs than apo E despite the lack of apo E [70]. While the study illustrates an interaction between VLDL and the peptide, additional structural data to quantify how much of the peptide interacts with VLDL would solidify the claim [70]. Additionally, the effect of apolipoprotein displacement is only seen on VLDL. However, does AEM-28 have the same ability to displace apo B-100, or do some lipid-bound domains of apo B-100 require further studies [70]?

## 6. Evaluation of AEM-28 in Preclinical Models

AEM-28 and its analogs were studied in diet-induced dyslipidemic, apo E-null, LDL-R null, and apo E/LDL-R double knockout mice. Studies aimed to establish the peptide’s ability to reduce plasma cholesterol and to observe whether the effect is via HSPG using heparinase/heparitinase treated mice [71]. Table 1 shows the methodology for each mouse strain used, as well as the amount of the peptide AEM-28 administered, and cholesterol levels before and after peptide administration [71]. In apo E-null mice on normal chow, the baseline cholesterol levels (Table 1) were highly elevated, and after peptide administration, cholesterol levels decreased to 51.5 mg/dL at 6 h and rebounded after 8 h [71]. The decrease in cholesterol was only observed in the VLDL and IDL/LDL fractions; it was not seen in the HDL fraction [71]. The observed decrease in atherogenic lipoproteins could suggest that AEM-28 has an innate selectivity for LDL/VLDL [71]. Additionally, the redistribution of the peptide in the rapid phase showed that AEM-28 is cleared from plasma in approximately 2 min [71]. From a pharmacokinetic perspective, this suggests that the peptide is effective immediately upon administration [71]. The half-life of AEM-28 in apo E-null mice also showed peptide clearance in approximately 90 min [71].

Peptide administration in C57BL/6J mice fed normal chow (Table 2) did not lead to a significant reduction in plasma cholesterol [71]. However, in mice fed the atherogenic diet, a rapid decrease in plasma cholesterol was observed (Table 2) [71]. These findings suggest that once a healthy mouse increases their atherogenic lipoprotein count via their diet, there are enough atherogenic lipoproteins for AEM-28 to interact with and clear from plasma [71]. Table 1 shows the methodology for LDL-R null mice as well as their plasma cholesterol reduction [71]. In both normal chow and Western diet-administered LDL-R null mice, AEM-28 did not significantly decrease the plasma cholesterol [71]. This suggests that human LDL and mouse LDL are inherently different, requiring further investigation [71]. It was shown meanwhile that the peptide associated with human LDL and VLDL, AEM-28, did not associate with mouse LDL from LDL-R null mice [71]. This indicates that the peptide must be associated with the human atherogenic lipoproteins for an observable effect on plasma lipoprotein clearance in vivo [71]. These discrepancies likely point toward significant structural differences between human and murine lipoproteins, which may account for the variation in AEM-28 binding affinity across species. Interestingly, administration of AEM-28 cleared plasma lipoproteins from LDLR/apo E double knockout mice fed on normal chow within 2 min; thus, the plasma cholesterol was reduced dramatically and remained low even after 6 h [71]. The reason for the non-binding of the peptide to LDL from LDL-R null mice is not clear; however, this demonstrated that human LDL is quite different from LDL-R null mouse LDL [71]. The observation seen in LDL-R null mice suggests that different animal models require different methodologies to replicate similar effects caused by AEM-28 [71].

The effect of AEM-28 on plasma cholesterol was investigated in female LDL-R null mice fed a Western diet [72]. In LDL-R null mice, chronic AEM-28 administration reduced plasma cholesterol, oxidative stress, macrophage accumulation, and atherosclerotic lesion development [72]. Lipoprotein profiles showed that the peptide reduced mostly VLDL-type lipoprotein, with some reduction in LDL, and an even smaller reduction in HDL [72]. While other mouse studies demonstrate plasma cholesterol reduction with only one administration of the peptide, chronic administration was necessary to see similar effects in LDLR-null mice [71,72]. This highlights a potential limitation in using LDLR-null mice as a model for AEM-28 [72]. Chronic AEM-28 usage also reduced plasma reactive oxygen species (ROS) compared to the control mice [72]. The reduction in ROS is an essential factor of AEM-28, due to ROS causing cellular stress, which increases monocyte chemotaxis into the intima, initiating an inflammatory pathway [15,72]. The peptide also inhibited the formation of atherosclerotic lesions in the aortic sinus of the mice [72]. The inhibition of lesion formation prevents the arterial tissue from hardening, maintaining elasticity and healthy circulation [15,72]. Additionally, the total macrophage content in the aortic sinuses was dramatically reduced in peptide-administered mice [72]. This feature of AEM-28 further supports alternative mechanisms in inhibiting atherosclerosis since the number of macrophages in an area afflicted by atherosclerosis can potentially reduce the inflammation [15,72]. Additionally, similar results were observed in both LDL-R null mice and apo E-null mice, suggesting that AEM-28 in chronic administration is not confined to one genetic/disease model for effective reduction in atherogenic lipoproteins, inflammatory biomarkers, and atherosclerotic lesions [66,72]. The chronic administration also supports the idea that certain disease states may need more frequent administration of the peptide or higher doses to see significant effects [72].

To determine the effect of AEM-28 on already existing lesions, 22-week-old apo E-null female mice were used (Table 3) [66]. Administration of AEM-28 significantly reduced plasma cholesterol and increased both PON-1 activity and PON-1 levels compared to control and 4F, an apo A-I mimetic peptide which has also been shown to reduce lesions in mouse models of atherosclerosis (Table 3) [66,73]. These results support the idea that AEM-28 can decrease inflammatory biomarkers as well as decrease lesions [66]. These reductions in inflammatory biomarkers and atherosclerotic lesions further support similar results seen in a previous study on female LDL-R null mice [66,72]. Reductions were seen in plasma cholesterol and in *en face* lesions, suggesting that AEM-28 reduces lesion size and reduces the progression of the existing lesion [66]. As seen in the previous experiments, plasma cholesterol and aortic *en face* lesions were reduced in the AEM-28 group [66]. AEM-28 showed similar results in increasing PON-1 activity to 4F [66]. An increase in PON-1 activity mediated by AEM-28 is beneficial, since the enzyme protects lipoproteins from oxidation, reducing lipid hydroperoxides in the body [66,74]. Additionally, AEM-28 reduced the HDL inflammatory index and the plasma inflammatory index [66]. These indices measure monocyte chemotaxis activity in cultured human aortic endothelial cells when given HDL or plasma, indicating pro- and anti-inflammatory responses [75]. The data in this model suggest a similar effect seen in female LDL-R null mice, where AEM-28 actively inhibits the formation of new atherosclerotic lesions, as well as increases anti-inflammatory enzymes such as PON-1 [66]. The capability of AEM-28 to lower the HDL inflammatory index suggests that HDL is being converted from a proatherogenic state to an atheroprotective state [66]. Additionally, while decreasing the plasma inflammatory index, this supports that the peptide can decrease LDL-mediated monocyte chemotaxis, decreasing the inflammatory response [66]. Understanding the duration of the antioxidant enzyme activity increase, as well as the extent of lesion reduction, will further provide information on the pleiotropic effects of AEM-28.

Additional studies were conducted on apo E-null mice and THP-1 Macrophages to evaluate the peptide’s pleiotropic effects on cellular reengineering and atheroprotective effects. The analog AEM-2 was used for this study; the apo E binding sequence converted the lysine on positions 3 and 6 to arginine (LRRLRRRLLR) linked to 18A with an acetylamino hexanoic acid (Ac-Aha) at the N-terminus [63]. These modifications enhanced the peptide’s inherent hydrophobicity and enhanced its binding ability to atherogenic lipoproteins [63]. The clearance of plasma cholesterol was evaluated in apo E-null mice [63]. Administration of AEM-2 showed enhanced clearance of cholesterol at 1 and 5 h: 80% and 82%, respectively [63]. A similar reduction in plasma cholesterol was observed in AEM-2 compared to AEM-28, suggesting that modifications to the peptide can increase plasma cholesterol clearance [63].

The peptide AEM-2 was next evaluated on THP-1 Macrophages to test its inflammatory response [63]. Macrophages were given AEM-2, then placed into LPS-media to observe an inflammatory response [63]. Cells given a low dose of AEM-2 did not show a significant reduction in inflammatory markers [63]. However, the cells administered a high dose of the peptide reduced IL-6 by 37% and TNFα by 41% [63]. Both biomarkers are associated with cardiovascular disease, and their reduction mediated by AEM-2 decreases the inflammation caused by these biomarkers [63,76]. LPS-induced mitochondrial oxidant production was observed with and without the peptide [63]. The peptide at a high dose showed a significant reduction in oxidant formation, as well as an increase in the Δψm [63]. This is critical since when the mitochondrial membrane depolarizes, this introduces Cytochrome C into the cytosol, leading to apoptosis [77]. At a high dose, the peptide also significantly reduced the release of Cytochrome C [63], therefore reducing apoptosis significantly, since Cytochrome C is not present in the cytosol [63,77]. The reduction in apoptosis in atherosclerosis can be beneficial depending on the cell type and may inhibit progression of the disease [78]. At both low and high doses of AEM-2, dramatic reductions in the activity of Caspase 9, Caspase 8, and Caspase 3 were observed [63]. The reductions in enzyme activity suggest that AEM-2 reduces Caspase-mediated apoptosis [63]. Next, autophagy within THP-1 cells was evaluated, since pathways associated with apoptosis and autophagy have been shown to crosstalk in some capacity [63]. A high dose of AEM-2 induced autophagic flux, suggesting that the peptide stimulates the induction and turnover of autophagosomes [63]. The variant, AEM-2, shows that it can induce anti-inflammatory and anti-apoptotic properties within THP-1 Macrophages [63]. Moreover, these effects observed by AEM-2 and potentially AEM-28 could reprogram certain cells to undergo different outcomes that are overall beneficial by clearing out oxidized and atherogenic lipoproteins, reducing the progression of inflammation and atherosclerosis [63].

Sepsis is a life-threatening infection caused by the release of bacterial lipopolysaccharide (LPS) in the bloodstream [64]. Researchers studied the effect of AEM-28 on LPS-induced inflammation using C57BL/6 mice. Two serotypes of *E. coli* were used because of their differences in LPS content: 055:B5 is a smooth serotype with a long O-chain, and 026:B6 has a short O-chain resembling that of the rough mutant [79]. In the control mice administered, elevated levels of plasma endotoxin activity were observed, leading to high levels of IL-6 and ROS [79]. Administration of the peptide dramatically reduced plasma endotoxin activity, IL-6, and ROS at 3 h [79]. This reduction in inflammatory markers further supports the notion that AEM-28 carries pleiotropic effects that may mediate inflammation [63,72,79]. Similar results were seen in older apo E-null mice [66,79]. At 27 h, AEM-28 reduced plasma SAA and ROS levels significantly in comparison to the control and 4F administered group [79]. The reduction in inflammatory biomarkers establishes one set of pleiotropic effects that has been shown in numerous experimental models [63,72,79]. An experiment using the O55:B5 serotype LPS, using similar parameters as the previous one, was performed [79]. Plasma endotoxin activity was inhibited by 93%, and plasma IFN*γ* was significantly reduced at 3 and 24 h [79]. The reduction in IFN*γ* suggests that AEM-28 interacts in some capacity with various immune cells such as mast cells and TH-1/17 cells [16,79]. However, more research is needed to evaluate AEM-28’s capacity to address the heterogeneity of immune cells involved in inflammation and atherosclerosis [16,79]. AEM-28 decreased plasma endotoxin activity similar to that of control mice with no peptide administration, and inhibited LPS-induced elevation of plasma ROS levels [79]. These observations suggest that AEM-28 carries various beneficial properties that can apply to various lipid-mediated disease states even if it is not associated with hypercholesterolemia [79]. An experiment was performed to determine the effect of the peptide post-LPS infection with AEM-28, administered three hours after LPS injection [79]. Thirty minutes after peptide injection, plasma endotoxin activity significantly decreased [79]. Sequential administrations of LPS and AEM-28 showed a dramatic reduction in plasma ROS levels [79]. With AEM-28 having a high lipid-binding affinity, a potential interaction between LPS and the peptide may be occurring, explaining the significant decrease in LPS-induced inflammation [79]. These studies support that AEM-28 reduces already existing inflammation in animals and thus inhibits LPS from activating downstream inflammation [79].

In patients suffering from insulin sensitivity or type 2 diabetes, it is common to see plasma lipid/lipoprotein abnormalities, which can lead to increased chances of coronary artery disease (CAD) [26]. Those suffering from lipid abnormalities and those who are type 2 diabetic/insulin-sensitive use statins and similar medications [26]. However, those therapeutics have their limitations as previously mentioned [26]. AEM-28 was administered to dyslipidemic C57BL6/J male mice to observe changes in insulin levels, blood glucose levels, and body weight [26]. AEM-28 administration showed increased plasma insulin levels and insulin sensitivity [26]. Additionally, plasma glucose levels and body weight were reduced [26]. While the effects observed are beneficial overall in this model, additional investigation is required to understand the type of interactions that may occur between the peptide and pancreatic cells that allow for an increase in insulin levels. Further testing would still be required on diabetic animal models to support AEM-28’s role in diabetes. Care should be used to extrapolate these preclinical results due to the fact that not all animal studies can be translated to humans. Additionally, further investigation using a mouse model that is both dyslipidemic and diabetic could support the claims.

New Zealand White (NZW) rabbits are highly susceptible to cholesterol-rich diets and develop hypercholesterolemia in two weeks, developing atherosclerosis [30]. NZW rabbits were fed a cholesterol-supplemented diet for 20 days and administered 3 mg (1 mg/mL in PBS) of AEM-28 via the ear vein [30]. Plasma samples from the rabbit before peptide administration were turbid with an off-white color. The peptide showed no effects on the plasma cholesterol levels at shorter time intervals [30]. However, 15 days after the first administration, the plasma from the rabbit was clear, and cholesterol was also significantly reduced [30]. These data support that AEM-28 in higher-order mammals takes longer to clear plasma cholesterol and highlights the need for further investigation on the effect of AEM-28 on other higher-order mammals such as Watanabe rabbits and monkeys [30].

The Watanabe Heritable Hyperlipidemic rabbit (WHHL) is a unique strain of rabbit that develops severe hypercholesterolemia and spontaneous atherosclerotic lesions even on the normal rabbit diet [80]. The rabbits possess a genetic deficiency in the LDL receptor, resembling human homozygous familial hypercholesterolemia (FH) [80]. This causes total cholesterol and LDL levels to be 8 to 14 times higher than in normal rabbits, making it a viable model for studying the effect of AEM-28 on FH [80]. The rabbit protocol for AEM-28 administration is given in Table 4 [49]. Single administration of AEM-28 produced a dose-dependent decrease in total plasma cholesterol compared to the control group [49]. This affirms that AEM-28 in higher-order mammals is dose-dependent, as seen earlier in various experimental models [49,67,70]. Table 4 shows that total plasma cholesterol reduction was dose-dependent [49]. In a separate experiment, 7.5 mg/kg was injected into male WHHL rabbits, and mean plasma triglyceride levels were reduced by 45% for up to 8 h but returned close to, but lower than, pre-administration levels [49]. These results indicated that the peptide is associated with VLDL and is able to reduce plasma cholesterol via the HSPG pathway [49]. Plasma analysis at varying time-points showed that ^125^I-labeled AEM-28 radioactivity was associated with VLDL, LDL, and HDL [49]. This was seen even when HDL levels were low; however, PON activity in HDL increased after peptide administration [49]. The increased PON activity was associated with a decrease in total plasma lipid hydroperoxide levels [49]. This increase in PON-1 activity suggests that AEM-28 increases enzyme activity quickly, which results in the decrease in lipid hydroperoxides [49]. The data in WHHL rabbits support the results seen in apo E-null mice: AEM-28 can increase PON-1 activity regardless of the animal model [49,66]. Table 5 and Table 6 show that AEM-28 was able to increase endothelium-dependent relaxation and was similar to control rabbits [49]. The increase in relaxation of the endothelium suggests that AEM-28 can allow arteries to dilate similar to healthy counterparts, an important observation in maintaining healthy arterial walls [49]. Furthermore, AEM-28 administration improved acetylcholine-induced relaxation by 24% [49]. This increase in endothelium relaxation via AEM-28 is beneficial since acetylcholine binding to the endothelium causes the release of nitric oxide, a vasodilator [49,81]. The effect of the peptide on the formation of ROS was also measured, such as O_2_^−^, which is a scavenger of nitric oxide [49]. AEM-28 reduced superoxide anion production in aortas by 68% [49]. The data from WHHL rabbits suggest that AEM-28 in higher-order mammals significantly reduces plasma cholesterol in a dose-dependent manner, as well as significantly reducing inflammatory markers [49].

AEM-28 reduced plasma cholesterol and exerted anti-inflammatory effects in mice and rabbits. To improve the efficacy of the peptide, further modifications were explored, including the addition of fatty acids to create AEM-28 analogs. The various analogs that were tested are given in Table 7, as well as their injection protocol for both apo E-null mice and monkeys [82]. The baseline plasma cholesterol for mice on a normal chow diet was 400 to 500 mg/dL; the peptides AEM-28, Ac-[R]hE18A-NH_2,_ and AEM-2 are evaluated in Table 7 [82]. AEM-2 was the most effective among the three peptides in reducing plasma cholesterol [82]. This is due to the acetylamino hexanoic acid (Ac-Aha) increasing the hydrophobicity of the peptide, allowing it to bury the peptide backbone further into the lipoprotein, increasing lipoprotein clearance [82]. Oct-[R]hE18A-NH_2_ and Myr-[R]hE18A-NH_2_ were tested in apo E-null mice fed a normal chow diet [82]. Oct-[R]hE18A-NH_2_ reduced plasma cholesterol by approximately 35%, whereas Myr-[R]hE18A-NH_2_ saw a 60% reduction in plasma cholesterol [82]. Similar results were seen in the Myr-AEM-28 analog compared to Myr-Ac-[R]hE18A-NH_2_ [82]. This showed that, by increasing the hydrophobicity of the peptide, an increase in plasma cholesterol reduction is also seen [82].

Based on these results, Myr-[R]hE18A-NH_2_ was studied in monkey models of dyslipidemia [82]. Monkeys on a cholesterol-supplemented Western diet for 12 weeks showed mean TC and LDL levels of 300 and 225 mg/dL, respectively [82]. Administration of 7.4 mg/kg resulted in the greatest reduction in plasma cholesterol, which was maintained for 3 days after administration and returned to the pre-administration level 7 days after administration [82]. A single administration of the peptide had lasting effects for three days, showing the enhanced effect of the fatty acid addition in terms of plasma cholesterol clearance in an animal model similar to humans [82].

The agarose electrophoretic mobility of isolated LDL from monkeys given 7.4 mg/kg of Myr-[R]hE18A-NH_2_ was decreased compared to LDL, and this was even seen after 24 h [82]. Again, these results support that the peptide could associate with LDL from a species closely related to humans, indicating lipid-binding ability prompting clinical testing [82]. There was also a reduction in apo B levels, which correlated with the reduction in LDL levels seen in Table 7 [82]. Additionally, seeing strong LDL clearance in a monkey model emphasizes the translational value of the peptide [82]. Interestingly, there was also a reduction in HDL and plasma apo A-I levels [82]. Why this reduction in HDL and apo A-I occurred requires further investigation [82]. Even after 7 days, atherogenic LDL-C was still significantly reduced with only one dose of the peptide [82]. With only one administration and lasting effects observed for 7 days, further investigation into the pharmacokinetics is required [82]. Interestingly, HDL levels rebounded rapidly and were equal to or exceeded the baseline after 3 days of administration [82]. The recovery of HDL levels following treatment may indicate favorable effects on lipoprotein remodeling; however, the underlying mechanisms remain unclear and require further investigation [82]. Additionally, Myr-[R]hE18A-NH_2_ was the best peptide in terms of reducing plasma cholesterol in both apo E-null mice and in monkeys, warranting the initiation of clinical trials [82]. Moreover, it would be beneficial to study these modified high-lipoprotein affinity analogs in mouse models where AEM-28 did not associate with LDL and decreased plasma cholesterol [82]. Lastly, it would have been pertinent for each acyl peptide analog, as well as AEM-28, to be used in the monkey model to understand the contribution of increasing hydrophobicity at the N-terminus for cholesterol clearance [82]. This lack of such investigations could have been due to the cost and practicality of using monkeys as a model.

## 7. Phase I Clinical Trial

AEM-28 showed a reduction in plasma cholesterol across numerous cell and hyperlipidemic animal models. The Phase I trial was initiated to determine if the peptide exhibited toxicity. Phase I studies were initiated by LipimetiX Development, LLC, and the trial was conducted in Australia [30,83]. The trial protocol consisted of a blended phase 1a/1b, single/multiple ascending dose (SAD/MAD) protocol with 52 participants [30,83]. The SAD portion of the study investigated the safety and tolerability of ascending doses of AEM-28 ranging from 0.032 to 3.54 mg/kg [30,83]. Healthy volunteers were placed into the SAD portion, and individuals with elevated cholesterol on statins were included in the MAD portion [30,83]. Overall, AEM-28 was well tolerated; at the highest doses, venous irritation was observed [30,83]. No significant adverse events were reported in either portion of the study [30,83]. The subjects within the MAD portion were given three doses, spaced 2 weeks apart, and each dose was escalated after the previous cohort received their second dose [30,83]. Venous irritation was noted at 3.54 mg/kg, and the MAD portion was terminated after the last cohort received their first dose [30,83]. It is possible to avert the irritation problem with a suitable formulation since the peptide has been shown to reduce plasma cholesterol when complexed with lipids [84]. Rapid and significant dose-dependent decreases in VLDL and total triglycerides were noted [30,83]. Patients in the MAD portion saw a 75% decrease in VLDL and total triglycerides compared to the baseline [30,83]. Preliminary results from the trial suggest that AEM-28 may induce a rapid response in lowering VLDL and total triglycerides in human subjects [30,83]. Although the trial aimed to study the safety and tolerability of the peptide administration in patients, the study yielded a significant reduction in atherogenic lipoproteins. Furthermore, no evidence of a neutralizing antibody response was observed [30,83]. Detailed immunogenicity methodology and anti-drug antibody testing procedures were not reported in publicly available trial records, limiting interpretation of the observed lack of neutralizing antibody responses [30,83]. Additionally, since detailed in silico analyses for immunogenic motifs and data regarding total anti-drug antibodies are unavailable, a direct comparison between the immunogenicity profile of AEM-28 with other clinically established peptide therapeutics is not presently feasible. Consequently, the full extent of the immune response to repeated administration of AEM-28 remains a limitation that warrants further investigation in future clinical studies. The peptide does exhibit a short half-life as well as rapid clearance of plasma cholesterol in various mouse models. However, in higher-order mammals, a slower rate of cholesterol reduction has been observed. In these models, the rate of peptide clearance from plasma has not been investigated. We hypothesize that the formation of stable peptide–lipid complexes may protect the peptide from immediate degradation, effectively creating a “reservoir” effect that extends its biological activity beyond its initial plasma half-life.

Further studies are required to optimize the dosage, route of administration, and formulation, as well as to fully evaluate safety and tolerability within hyperlipidemic populations. To date, clinical development has concluded following Phase I, as funding constraints precluded LipimetiX from advancing to subsequent trial phases.

## 8. Conclusions

**Figure 4:**
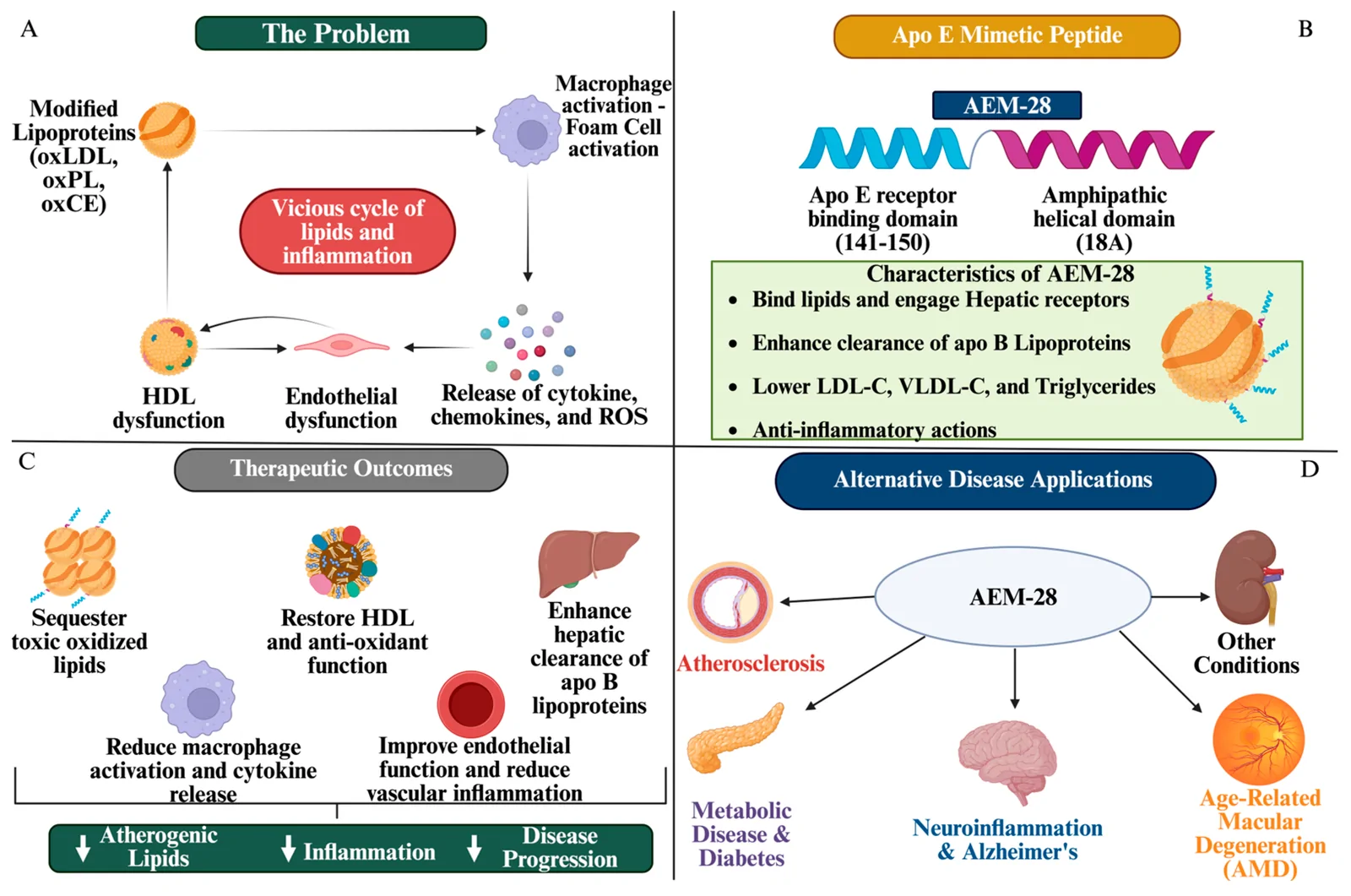
Panel (**A**) discusses the issue of oxidized LDL and the cycle of inflammation that is caused by oxidized LDL. Panel (**B**) discusses the components of AEM-28 as well as its characteristics. Panel (**C**) describes the outcomes that come with AEM-28 seen in studies mentioned in the paper. Panel (**D**) shows alternative disease states that could be further investigated with AEM-28. Created in BioRender. Patel (2026) https://BioRender.com/.

AEM-28 is a dual-domain peptide that combines the binding domain of apo E and is covalently linked to an apo A-I mimetic, 18A. Conventional therapeutics focus on inhibiting cholesterol biosynthesis or LDL-R degradation. AEM-28’s mechanism of action focuses on attaching multiple copies of the peptide to atherogenic lipoproteins and clearing them via the HSPG receptors present abundantly on hepatocytes. The clearance of atherogenic lipoproteins in this manner is a novel feature of AEM-28 and has been supported via multiple experimental models. This novel characteristic of lipoprotein clearance has not been seen in other compounds and indicates a first in class in terms of a peptide therapeutic in the application of hypercholesterolemia and atherosclerosis. Additionally, the pleiotropic effects observed in AEM-28 allow for the peptide to be applied to various disease states such as diabetes and others mentioned in Figure 4. The unique characteristics of AEM-28 position the peptide to address the therapeutic benefit to hypercholesterolemia, cardiovascular disease, and atherosclerosis. To advance AEM-28 toward clinical viability, several critical limitations must be addressed, most notably regarding formulation and precise structural interaction. For AEM-28 to have success as a therapeutic, improved formulation may be required to mitigate the venous irritation noted during clinical testing. Additionally, further research is needed on analogs of AEM-28 that outperformed the parent AEM-28 peptide, such as the fatty acid analogs, potentially in clinical testing and observing if it carries the same pleiotropic effects. Furthermore, while AEM-28 effectively reduces cholesterol, the precise nature of its interaction with lipoproteins remains a key unresolved question. Future structural analysis using electron microscopy will determine whether AEM-28 facilitates the formation of a synthetic lipid particle or binds to existing core apolipoproteins. Resolving this critical difference in mechanism will clarify if or how the peptide displaces bound proteins and will better inform the optimization of its therapeutic efficacy. Lastly, as new research provides insight into the heterogeneity of the immune system in atherosclerosis, exploring AEM-28’s effect on specific cell types may better explain its pleiotropic and anti-inflammatory potential. Ultimately, the unique properties of AEM-28 provide a compelling foundation for a new generation of peptide therapeutics capable of addressing not only plasma cholesterol but the broader spectrum of dyslipidemic and inflammatory conditions.

## Figures and Tables

**Table 1:** Target, lipoprotein reduction, anti-inflammatory effect, and dose range for therapeutics used for patients with hypercholesterolemia. Created in BioRender. Patel (2026). https://BioRender.com/ [27,28,29,30].

Therapy	Target	Atherogenic Lipoprotein Reduction	Anti-Inflammatory Effect	Dosing Limit
Statins	HMG-CoA Reductase	30–63%	• Reduction in inflammatory chemokines/cytokines • Reduction in reactive oxygen species • Plaque stabilization	10–80 mg
Alirocumab/Evolocumab	PCSK9 protein	45–65%	• Reduction in TLR4-associated cytokine cascade • Reduction in Osteopontin, Osteoprotegerin, and Metalloprotease-9 • Reduction in reactive oxygen species • Plaque stabilization	75–420 mg
Inclisiran	PCSK9 mRNA	45–65%		284 mg
AEM-28	LDL/VLDL	72%	• Reduction in reactive oxygen species • Increase in PON-1 amount/activity • Reduction in TNF-α/IL-6 inhibition of LPS-mediated inflammation • Increase in endothelium-dependent relaxation • Reduction in HDL/plasma inflammatory index	3.54 mg/kg

**Table 2:** Methodology of the study, displaying the type of diet, baseline cholesterol, peptide administration, and the cholesterol reduction after injection at several time-points. Created in BioRender. Patel (2026). https://BioRender.com/.

Mouse Strain	Type of Diet	Baseline Cholesterol (mg/dL)	Amount of AEM-28 Injected	Cholesterol After Injection (mg/dL)
Female C-57 Black 6C	Normal chow	71.6 ± 4.7	100 µg in 100 µL Sterile PBS	68.1 ± 4.2 at 2 min & 82.8 ± 7.3 at 6 h
Female C-57 Black 6C	Western diet	187.9 ± 15.6	100 µg in 100 µL Sterile PBS	88.3 ± 8.2 at 2 min & 111.3 ± 7.7 at 6 h
apo E null	Normal chow	379.3 ± 15.7	100 µg in 100 µL Sterile PBS	51.5 ± 2.0 at 6 h, 148.2 ± 27.1 at 8 h, 268.7 ± 17.0 at 24 h
LDL-R Null	Normal chow	249.3 ± 12.2	100 µg in 100 µL Sterile PBS	252.5 ± 8.9 at 2 min & 276.5 ± 9.5 at 6 h
LDL-R Null	Western diet	753.3 ± 29.6	100 µg in 100 µL Sterile PBS	777.4 ± 32.7 at 2 min & 705.4 ± 41.5 at 6 h

**Table 3:** Protocol for older apo E-null mice with effects observed. Created in BioRender. Patel (2026). https://BioRender.com/.

Time of AEM-28 Injection	Amount of AEM-28 Injected	Time of Blood Samples Taken	Baseline Plasma Cholesterol (mg/dL)	Plasma Cholesterol After AEM-28 Injection (mg/dL)	Baseline PON-1 Activity (PON Units)	PON-1 Activity After AEM-28 Injection (PON Units)	En Face Lesion Area % Before AEM-28 (% Surface Area)	En Face Lesion Area % After AEM-28 (%Surface Area)
2, 5, 15, 30, 45 min 1, 2, 4, 6, 24 h	100 µg in Saline	2, 5, 15, 30, 45 min 1, 2, 4, 6, 24 h	354 ± 26	263 ± 23	1.190 ± 0.062	1.462 ± 0.062	14.70 ± 1.56	6.35 ± 1.06

**Table 4:** Dose-dependent reduction in plasma cholesterol after administration of AEM-28 in WHHL rabbits. Created in BioRender. Patel (2026). https://BioRender.com/.

Rabbit Strain	Diet	AEM-28 Concentration (mg/kg)	Mean Plasma Cholesterol Reduction %
Male Homozygous Watanabe Heritable Hyperlipidemic	Normal chow	2.5	14.91
		7.5	41.39
		15	58.1

**Table 5:** Dramatic effects of administration of 7.5 mg/kg of AEM-28 on plasma cholesterol. Created in BioRender. Patel (2026). https://BioRender.com/.

Rabbit Strain	Diet	AEM-28 Concentration (mg/kg)	Baseline Plasma Cholesterol (mg/dL)	Plasma Cholesterol After AEM-28 (mg/dL)	% Plasma Cholesterol Reduction
Homozygous WHHL	Normal chow	7.5	562.9 ± 29.0	287.7 ± 22.0	51.11

**Table 6:** Results showing the effects of AEM-28 on endothelium-dependent relaxation. Created in BioRender. Patel (2026). https://BioRender.com/.

Rabbit Strain	Diet	AEM-28 Concentration (mg/kg)	Endothelium-Dependent Relaxation %
Homozygous WHHL	Normal chow	15	95 ± 6
Homozygous WHHL	Normal chow	N/A	71 ± 7
Normolipidemic	Normal chow	N/A	100 ± 3

**Table 7:** Enhanced cholesterol clearing effects of various acyl-analogs of AEM-28 with their sequence and the protocol for administration. Created in BioRender. Patel (2026). https://BioRender.com/.

Peptide	Peptide Sequence	Peptide Administration (mg/kg)	Diet Administration	≈% Plasma Cholesterol Reduction
Ac-hE18A-NH_2_	Ac-LRKLKRRLRLR-18A-NH_2_	2.5–5.0 in apo E null mice; 7.5 in apo E null mice	apo E null mice—standard or Western diet	apo E null mice on standard diet—40
Ac-[R]hE18A-NH_2_	Ac-LRRLRRRLLL-18A-NH_2_	2.5–5.0 in apo E null mice	apo E null mice—standard or Western diet	apo E null mice on standard diet—45
Ac-Aha-[R]hE18A-NH_2_ (AEM-2)	Ac-Aha-LRRLRRRLLL-18A-NH_2_	2.5–5.0 in apo E null mice; 7.5 in apo E null mice	apo E null mice—standard or Western diet	apo E null mice on standard diet—80; apoE null mice on Western diet—80
Oct-[R]hE18A-NH_2_	Octanyl-LRRLRRRLLL-18A-NH_2_	2.5–5.0 in apo E null mice	apo E null mice—standard or Western diet	apo E null mice on standard diet—45
Myr-hE18A-NH_2_	Myristyl-LRKLKRRLRLR-18A-NH_2_	2.5–5.0 in apo E null mice	apo E null mice—standard or Western diet	apo E null mice on standard diet—60
Myr-[R]hE18A-NH_2_	Myristyl-LRRLRRRLLL-18A-NH_2_	2.5–5.0 in apo E null mice; 7.5 in apoE null mice; 1, 3, 5, or 7.4 in monkey	apo E null mice—standard or Western diet; monkeys—atherogenic diet	apo E null mice on standard diet—60; apo E null mice on Western diet—90; Monkey on atherogenic diet—80

## Data Availability

No new data were created or analyzed in this study. Data sharing is not applicable to this article.

## Abbreviations

The following abbreviations are used in this manuscript:

HDL	High-Density Lipoprotein
LDL	Low-Density Lipoprotein
VLDL	Very-Low-Density Lipoprotein
AEM-28	Apolipoprotein E Mimetic-28
AEM-2	Apolipoprotein E Mimetic-2
PON-1	Paraoxonase 1
LDL-R	LDL Receptor
LRP	Low-Density Lipoprotein Receptor-Related Protein
HSPG	Heparin Sulfate Proteoglycans
